# Parallel PI3K, AKT and mTOR inhibition is required to control feedback loops that limit tumor therapy

**DOI:** 10.1371/journal.pone.0190854

**Published:** 2018-01-22

**Authors:** Anuja Sathe, Géraldine Chalaud, Immanuel Oppolzer, Kit Yeng Wong, Margarita von Busch, Sebastian C. Schmid, Zhichao Tong, Margitta Retz, Juergen E. Gschwend, Wolfgang A. Schulz, Roman Nawroth

**Affiliations:** 1 Department of Urology, Klinikum rechts der Isar, Technische Universität München, Munich, Germany; 2 Department of Urology, Medical Faculty, Heinrich Heine University Duesseldorf, Düsseldorf, Germany; University of South Alabama Mitchell Cancer Institute, UNITED STATES

## Abstract

Targeting the PI3K pathway has achieved limited success in cancer therapy. One reason for the disappointing activity of drugs that interfere with molecules that are important player in this pathway is the induction of multiple feedback loops that have been only partially understood. To understand these limitations and develop improved treatment strategies, we comprehensively characterized molecular mechanisms of PI3K pathway signaling in bladder cancer cell lines upon using small molecule inhibitors and RNAi technologies against all key molecules and protein complexes within the pathway and analyzed functional and molecular consequences. When targeting either mTORC1, mTOR, AKT or PI3K, only S6K1 phosphorylation was affected in most cell lines examined. Dephosphorylation of 4E-BP1 required combined inhibition of PI3K and mTORC1, independent from AKT, and resulted in a robust reduction in cell viability. Long-term inhibition of PI3K however resulted in a PDK1-dependent, PIP3 and mTORC2 independent rephosphorylation of AKT. AKT rephosphorylation could also be induced by mTOR or PDK1 inhibition. Combining PI3K/mTOR inhibitors with AKT or PDK1 inhibitors suppressed this rephosphorylation, induced apoptosis, decreased colony formation, cell viability and growth of tumor xenografts. Our findings reveal novel molecular mechanisms that explain the requirement for simultaneous targeting of PI3K, AKT and mTORC1 to achieve effective tumor growth inhibition.

## Introduction

Frequent hyperactivation and deregulation of the phosphoinositide 3-kinase (PI3K)/ AKT/ mammalian target of rapamycin (mTOR) pathway in cancer has made it one of the most investigated therapeutic targets in tumor therapy. Class IA PI3Ks, consisting of a p85 regulatory subunit and a p110 catalytic subunit, with the isoforms p110α, p110β, p110γ and p110δ, phosphorylate phosphatidylinositol-4, 5 bisphosphate (PI-4,5-P2) to phosphatidylinositol- 3,4,5-trisphosphate (PIP3). This reaction is reversed by the protein phosphatase and tensin homolog (PTEN) [1,2]). PIP3 initiates further signaling cascades by recruiting molecules such as AKT and PDK1 via their pleckstrin homology domains. AKT, a serine-threonine kinase, is functionally activated by phosphorylation at two distinct amino acid residues, threonine 308 and serine 473, by PDK1 and mTORC2, respectively. mTORC2 is a protein complex including the kinase mTOR and rapamycin-insensitive companion of mTOR (Rictor) [3]. Phosphorylated AKT in turn has the potential to regulate multiple downstream effectors and signaling pathways that are involved for example in cell proliferation, apoptosis, migration, and metabolism [4].

One downstream effector is the mTORC1 protein complex, which also contains the kinase mTOR, together with regulatory-associated protein of mTOR (Raptor), mLST8, Deptor and proline rich AKT substrate 40 kDa (PRAS40) [3]. Two important mTORC1 substrates are ribosomal protein S6 kinase beta-1 (S6K1) and eukaryotic translation initiation factor 4E-binding protein 1 (4EBP1). Phosphorylated S6K1 promotes the translation of 5’-terminal oligopyrimidine mRNAs while phosphorylation of 4E-BP1 prevents its binding to eIF4E and increases cap-dependent translation, thus controlling cellular protein synthesis and cell growth [5]. In this process, phosphorylation of the aminoacid residues Thr37/46, Ser65 and Thr70 in 4E-BP1 are essential [6].

The activity of mTORC1 has multiple levels of contro [7]. Raptor recruits substrates, including S6K1 and 4E-BP1, via their Tor signaling (TOS) motifs. It thus acts as a scaffolding molecule and directs the catalytic activy of mTORC1 [8]. AKT can stimulate mTORC1 activity by GTP-bound Rheb by regulating its GTPase activating protein (GAP) activity via phosphorylation of tuberous sclerosis complex 2 (TSC2) [7]. Activation of mTORC1 is further regulated by PRAS40 by competitive binding of its own TOS motif to Raptor. This inhibition can be reversed by PRAS40 phosphorylation at distinct sites by AKT and mTOR.

The first agents to target the PI3K pathway were rapamycin analogues (rapalogs), which bind to the protein FKBP-12 that complexes with mTOR, and thus allosterically inhibit mTORC1 activity [9]. These drugs have shown potential for the treatment of renal cell carcinoma, mantle cell lymphoma and neuroendocrine tumors which has fueled the development of additional classes of PI3K pathway inhibitors targeting all or specific PI3K isoforms, AKT, mTOR, or both PI3K and mTOR [10,11]. However, success in clinical trials has been lacking so far, with FDA approval granted only for the use of a PI3K δ inhibitor in chronic lymphocytic leukemia (CLL) [12]. Preclinical studies have demonstrated that inhibitors of the PI3K pathway can induce signaling feedback loops limiting their anti-tumor effects. For instance, rapalogs lead to increased AKT and ERK phosphorylation whereas dual PI3K/mTOR inhibitors lead to overexpression of different receptor tyrosine kinases [13]. Also, adaptive signaling responses after PI3K**α i**nhibition that increase PIP3 synthesis and AKT phosphorylation or enable SGK1-mediated mTORC1 activation have been recently described [14,15]. Successful therapeutic targeting of PI3K signaling thus requires a thorough understanding of the biochemical effects of PI3K pathway inhibition as well as effective drug combination strategies to overcome feedback loops limiting efficacy.

The PI3K pathway is overactive in around 72% of metastatic urothelial bladder cancer patients, making it an attractive target for therapy [16]. Currently, the average survival of these patients is only 12–14 months due to limited progress in therapy development since more than three decades, with PD1/PD-L1 immunotherapy only recently approved as second line therapy [17,18]. Clinical trials with rapalogs and other target therapies in bladder cancer have also demonstrated limited efficacy [19–22]. Our previous investigations have revealed that inhibition of both S6K1 and 4E-BP1 is necessary to efficiently suppress bladder cancer cell growth and proliferation. Rapalogs and AKT inhibitors, however, result in only S6K1 but not 4E-BP1 dephosphorylation, with a limited anti-proliferative effect. On the other hand, dual PI3K/mTOR inhibitors achieve dephosphorylation of both S6K1 and 4E-BP1, prohibiting cell cycle progression, but induce rephosphorylation of AKT after long term treatment [23,24]. In this study, by using different types of PI3K pathway inhibitors and knockdown of selected molecules in the pathway we demonstrate that parallel inhibition of mTORC1 and PI3K is crucial for 4E-BP1 dephosphorlyation, independently of AKT signaling. Furthermore, we describe a novel PI3K/PDK-1 dependent but PIP3 independent feedback loop resulting in AKT rephosphorylation upon long term PI3K inhibition that might limit therapy efficacy. This feedback loop can be overcome by parallel inhibition of PI3K/AKT and mTORC1 to efficiently block PI3K signaling, induction of apoptosis and tumor growth in bladder cancer.

## Materials and methods

### Cell lines and cell culture

Cell lines were maintained as early passages of subconfluent cultures in RPMI or DMEM (Biochrom AG, Berlin, Germany) at 5% or 10% CO2, respectively, supplemented with 10% FBS (Biochrom AG) and 1% NEAA (Biochrom AG). RT112 and 647V from the Leibniz institute German collection of microorganisms and cell cultures (Braunschweig, Germany), UMUC3, J82 and T24 were obtained from the American type culture collection (Manassas, VA, USA), 639 V and VmCUB1 were a kind gift from Professor Dr WA Schulz (Heinrich-Heine-University, Düsseldorf, Germany) and 253J were kindly provided by Professor Dr G. Unteregger (University of Saarland, Homburg/Saar, Germany). Cell lines were authenticated by short tandem repeat profiling and tested for mycoplasma using PCR as described previously [24,25].

### Small molecule inhibitors

Stock solutions of NVP-BEZ235, RAD001, NVP-BKM120 and GSK2334470 (Selleckchem, Munich, Germany), MK-2206 and INK128 (Active Biochem, Bonn, Germany), PIK-90 (Merck Chemicals GmbH, Darmstadt, Germany), NU7441 and KU60019 (Tocris Bioscience, Bristol, United Kingdom) were prepared in DMSO. Working concentrations were freshly prepared in medium with control corresponding to highest DMSO concentration.

### Oligonucleotides, transfection and transduction

Cells were transfected with 10 nM siRNA oligonucleotides against Raptor (CCCGUCGAUCUUCGUCUACGA), Rictor (UACGAGCGCUUCGAUAUCUCA) (Qiagen, Hiden, Germany) or negative control stealth RNAi high GC duplex #2 (Life Technologies, Darmstadt, Germany) using Lipofectamine RNAimax (Life Technolgies) per manufacturer’s instructions. Transduction of mTOR or control shRNA adenovirus was performed as described previously [23].

### Cell viability, apoptosis and cell cycle assays

CellTiter-Blue cell viability assay, caspase-Glo 3/7 assay (Promega Corporation) and click-it EdU Alexa Fluor 488 flow cytometry cell cycle analysis (Life Technologies) were performed as described [25].

### Clonogenic assay

150 RT112 cells were seeded in a 6-well format and treated with indicated inhibitors every 48 or 72 hours over a period of 12 days. Colonies were then fixed using 6% v/v glutaraldehyde and stained with 0.5% w/v crystal violet as described previously [26].

### Immunoblotting

Protein lysates were extracted and separated using SDS-PAGE and membranes were probed using primary antibodies (Cell Signaling Technology, Beverly, MA, USA) against pAKT Thr 308, pAKT Ser 473, total AKT, pS6K1 Thr 389, total S6K1, p4EBP1 Thr 37/46, p4EBP1 Ser65, total 4E-BP1, GAPDH, Raptor, Rictor, mTOR [23]. Chemiluminescence was analyzed on X-ray films or the ChemiDoc XRS+ system/Quantity One 1-D software (Bio-Rad Laboratories GmbH, Munich, Germany).

### PIP3/PI(4,5)P2 quantification

PIP3 and PI(4,5)P2 level quantification and normalization was performed using ELISA kits (Echelon Biosciences Inc, Salt lake city, UT, USA) using manufacturer’s protocol and as described previously [15].

### Chorioallantoic membrane (CAM) assay

The CAM assay was performed as described previously with seeding of 2 million cells on embryonic day (ED) 9, topical treatments on ED 11 and 13 and tumor harvesting on ED 15. Concentrations were calculated for the total embryo blood volume [24].

### Immunohistochemistry (IHC)

IHC and Ki-67 staining quantification was performed as described previously [24].

### Statistical analysis

Control or treatment conditions were compared using unpaired Student’s T test in Microsoft Excel. Determination of synergy was performed using Compusyn software (ComboSyn, Inc., Parasmus, NJ, USA) [27].

## Results

### PI3K/mTOR inhibitors control S6K1 and 4E-BP1 dephosphorylation and cell viability

**Fig 1:**
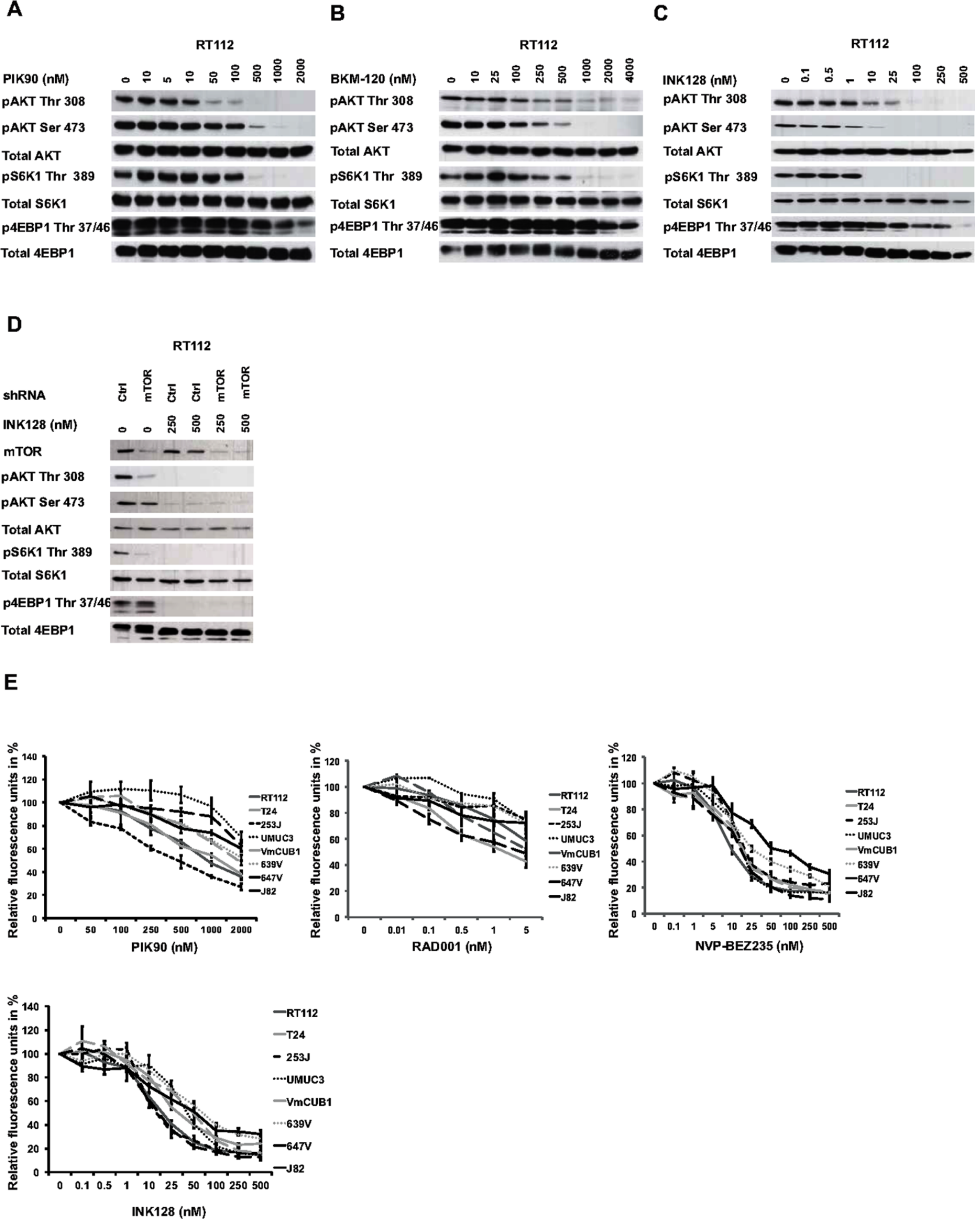
Effect of PI3K and PI3K/mTOR inhibitors on bladder cancer cell lines. (A-C) Cells were treated with indicated concentrations of PIK-90, BKM-120 or INK128 for 1 hour and immunoblotting was performed on lysates with the denoted antibodies. (D) Cell lysates and immunoblotting were performed with inhibitor or control treated cells for 1 hour after transduction with control (ctrl) or mTOR shRNA for 72 hours. (E) Cells were treated with respective inhibitors using indicated concentrations for 72 hours and cell viability assay was performed. Results indicate the mean +/- standard error of relative cell fluorescence in arbitrary units expressed as a percentage of control. All results are representative of at least three independent experiments.

Understanding regulation of 4E-BP1 is essential for targeting the PI3K signaling pathway for tumor therapy [23]. We extended previous data, showing that the mTORC1 inhibitor Everolimus (RAD001) robustly reduced S6K1 phosphorylation level. However, 4E-BP1 phosphorylation at Thr37/46 was not or only partially affected at high concentration and Ser65 phosphorylation level of 4E-BP1 was only downregulated at high concentration in the non-responding cell lines 253J and 647V S1A Fig [23,28]. Cell viability was affected by 20 to 50% Fig 1E. We next tested the effects of PI3K inhibition on 4E-BP1 phosphorylation using the pan-PI3K inhibitors PIK-90 and NVP-BKM120 [29,30]. These inhibitors dose-dependently diminished AKT phosphorylation at concentrations above 50–100 nM, accompanied by a parallel reduction in S6K1 phosphorylation and 10–50% effects on cell viability Fig 1A, 1B and 1E and S2A and S2B Fig. 4E-BP1 dephosphorylation was observed only at higher non-specific concentrations. When using the dual PI3K/mTOR inhibitor NVP-BEZ235, which targets the kinase activities of both PI3K (IC50 4–75 nM) as well as mTOR (IC50 20 nM), dephosphorylation of S6K1 was achieved with 5 Nm S3A Fig [23,31]. The mTOR kinase inhibitor INK128 decreased AKT and S6K1 phosphorylation at concentrations between 1–10 nM Fig 1C and S3B Fig. With both inhibitors, diminished 4E-BP1 phosphorylation was evident at almost 10-fold higher concentrations together with a 60–90% reduction in cell viability in all cell lines tested Fig 1E. Possible off-target effects of NVP-BEZ235 resulting in 4E-BP1 dephosphorylation were excluded by using specific inhibitors against ATM (KU60019) and DNA-PK (NU7441), as also PDK-1 (GSK2334470) S3C Fig [32].

mTOR silencing using an shRNA revealed that mTOR by itself only induced AKT and S6K1 dephosphorylation Fig 1D. INK128 treatment of cells with silenced mTOR expression continued to result in 4E-BP1 dephosphorylation. This indicates that either INK128 has an additional effect on PI3K activity or that remaining very low expression level of mTOR are sufficient to maintain 4E-BP1 phosphorylation.

### Combined inhibition of PI3K and mTORC1 results in 4E-BP1 dephosphorylation

**Fig 2:**
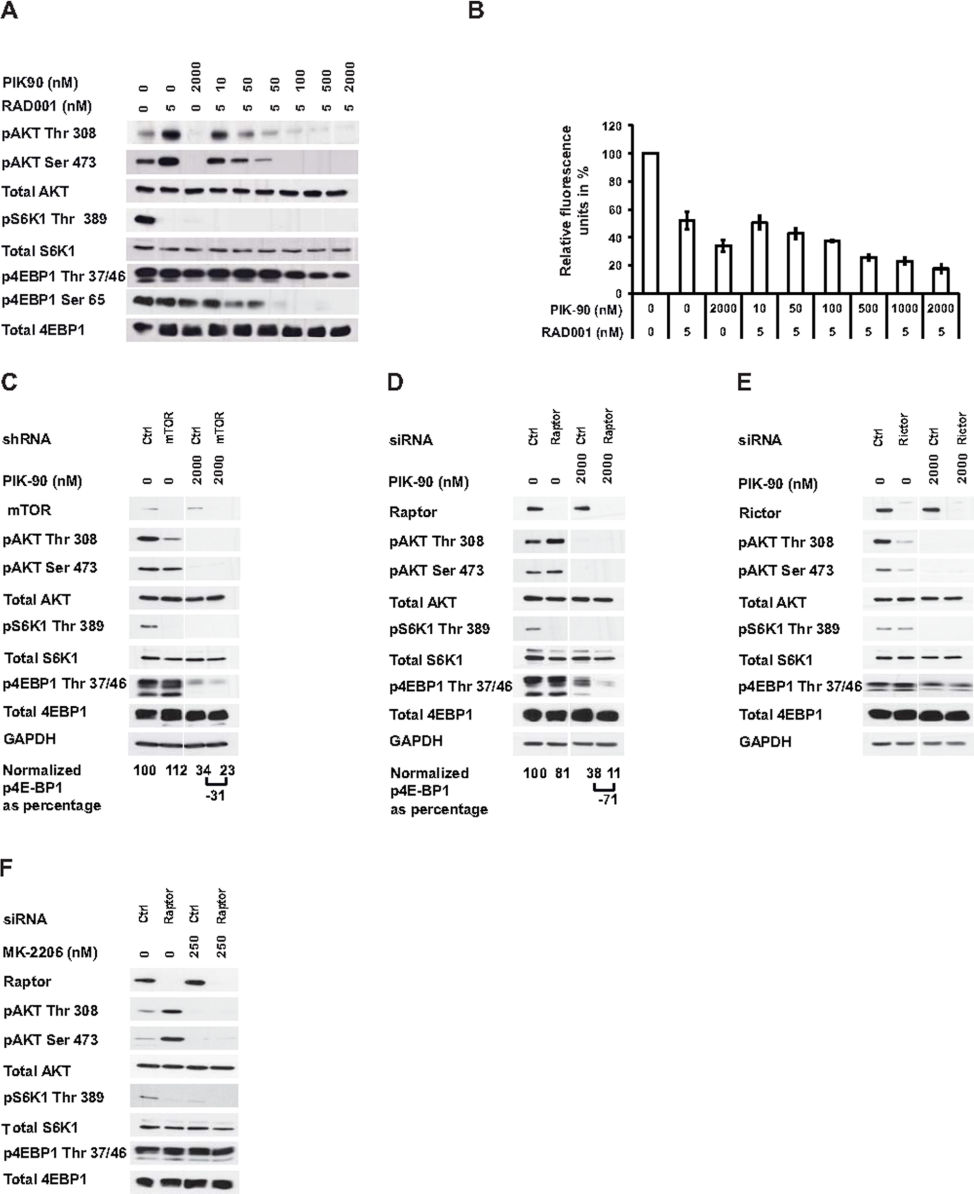
PI3K and mTORC1 regulate phosphorylation of 4E-BP1. Cells were treated with respective inhibitors at the indicated concentrations for 1 hour and immunoblotting was performed on lysates with the denoted antibodies (a) or cell viability was detected after 72 hours of treatment. Results indicate the mean +/- standard error of relative cell fluorescence in arbitrary units expressed as a percentage of control from three independent experiments. (c) Cells were transduced with control (ctrl) or mTOR shRNA for 72 hours, or (d-f) transfected with control (ctrl), Raptor or Rictor siRNAs for 48 hours. Cells were then treated with control or PIK-90 or MK-2206 at the indicated concentrations for 1 hour and immunoblotting was performed on lysates with the denoted antibodies. Normalized p4E-BP1 Thr 37/46 was calculated as percentage of control. Results are representative of at least three independent experiments.

To examine the underlying molecular mechanisms of 4E-BP1 regulation, we combined RAD001 with increasing concentrations of PIK-90. This treatment decreased 4E-BP1 phosphorylation at specific concentrations in all the cell lines examined Fig 2A and S4 Fig. Cell viability was reduced by 70–80% using this combination Fig 2B and S4B Fig. Combination of PIK-90 with an mTOR shRNA resulted in a 77% decrease in 4E-BP1 phosphorylation Fig 2C.

To determine whether mTORC1 or mTORC2 contributes to the observed effect, expression of either Raptor or Rictor was suppressed by specific siRNAs in combination with PIK-90. Raptor silencing decreased S6K1 phosphorylation and induced AKT hyperphosphorylation with only a 19% decrease in 4E-BP1 dephosphorylation Fig 2D but the combination with PIK-90 resulted in an 89% reduction in 4E-BP1 phosphorylation. Silencing the expression of Rictor resulted in diminished AKT phosphorylation without affecting 4E-BP1 phosphorylation Fig 2E. Importantly, combination of the allosteric AKT inhibitor MK-2206 that dephosporylates AKT and S6K1 with Raptor siRNA also failed to induce dephosphorylation of 4E-BP1 Fig 2F [24].

### Long-term PI3K/mTOR inhibition results in AKT rephosphorylation

**Fig 3:**
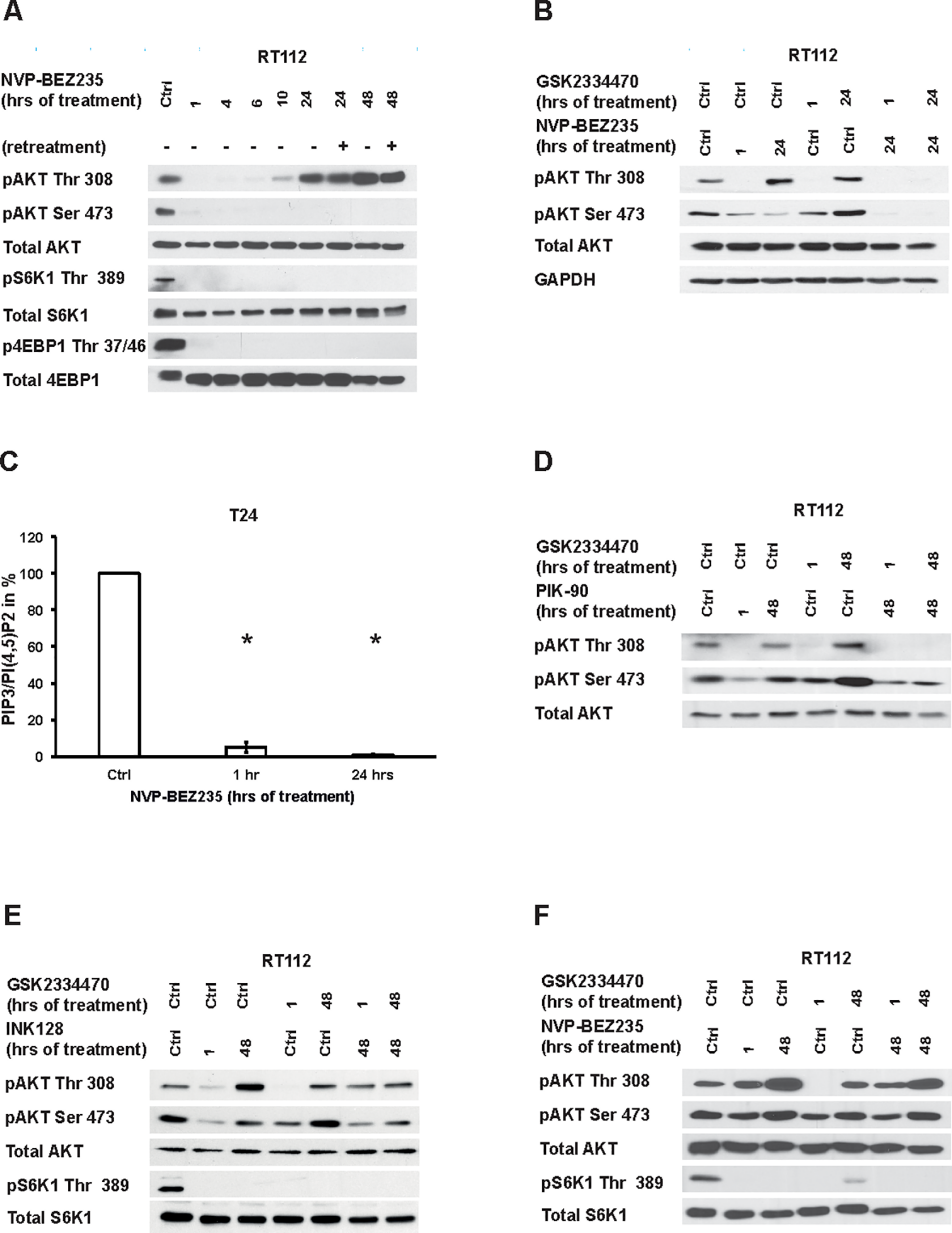
Inhibition of PI3K results in a positive feedback loop on AKT. RT112 cells were treated with 200 nM NVP-BEZ235 for the indicated duration or with control (ctrl). (A) Cells that were treated for 24 and 48 hours were additionally retreated with the same concentration for 1 hour (indicated by +, no retreatment indicated by -) and immunoblotting was performed with the respective antibodies (B) NVP-BEZ235 or control treatment was combined with 500 nM of GSK2334470 for the indicated duration or with control (ctrl). Immunoblotting was performed with the indicated antibodies. Results are representative of at least three independent experiments. (C) T24 cells were treated with control (ctrl) or 100 nM of NVP-BEZ235 for 1 or 24 hours. PIP3 and PI(4,5)P2 were extracted and the quantity was determined by ELISA. Results represent the mean +/- standard deviation of triplicate wells and indicate the PIP3/PI(4,5)P2 relative to control expressed as percentage. Results are representative of two independent experiments. * indicates p < 0.05 (unpaired Student’s T-test). (D-F) Cells were treated with control (ctrl), 500 nM GSK2334470, 500 nM PIK-90, 25 nM INK128 or 10 nM NVP-BEZ235 for the indicated duration and immunoblotting was performed on lysates with the indicated antibodies. Results are representative of at least three independent experiments.

NVP-BEZ235 treatment caused dephosphorylation of AKT at Thr 308 that recovered at 4–6 hours and was hyperphosphorylated at 24–48 hours after treatment Fig 3A and S5A Fig [23]. AKT at Ser 473, S6K1 and 4E-BP1, remained persistently dephosphorylated. Re-treatment of cells one hour before harvesting had no major effect on the rephosphorylation indicating no loss of inhibitor activity.

We then combined NVP-BEZ235 with the PDK1 inhibitor GSK2334470 to examine whether PDK-1 is responsible for the AKT rephosphorylation Fig 3B and S5B Fig. GSK2334470 monotherapy resulted in AKT dephosphorylation at Thr 308 within one hour of treatment but a hyperphosphorylation at both Thr 308 and Ser 473 was observed as described before [33]. However, combining GSK2334470 with NVP-BEZ235 successfully suppressed this rephosphorylation of AKT.

Recruitment of PDK1 to the cell membrane depends on generation of PIP3. In order to test PIP3 level, we used a previously validated ELISA based quantification of PIP3 [15]. The PIP3/PIP2 ratio decreased by 95% after 1 hour of and was not restored 24 hours after NVP-BEZ235 treatment Fig 3C indicating that PIP3 is not essential for the PDK-1 dependent rephosphorylation of AKT.

To further examine the role of PI3K or mTOR on inducing rephosphorylation of AKT Thr308, we first treated cells with a combination of PIK-90 and GSK233470 that successfully suppressed AKT rephosphorylation Fig 3D and S5C Fig. Next, we treated cells with a low dose of INK128 or NVP-BEZ235 Fig 3E and 3F and S5D and S5E Fig to inhibit mTOR but not PI3K. INK128 treatment caused dephosphorylation of S6K1 and AKT at predominantly Ser 473 within 1 hour. However, at 24 hours AKT was rephosphorylated at both Ser and Thr residues. The Thr308 rephosphorylation could only be partially suppressed by a combination of PDK1 and mTOR inhibitors. When using NVP-BEZ235, no dephosphorylation of AKT but only of S6K1 was observed at 1 hour. Interestingly, AKT became hyperphosphorylated after long-term treatment, which could not be prevented by additional PDK1 inhibition.

### Combination of dual PI3K/mTOR and AKT inhibitors reduces tumor growth

**Fig 4:**
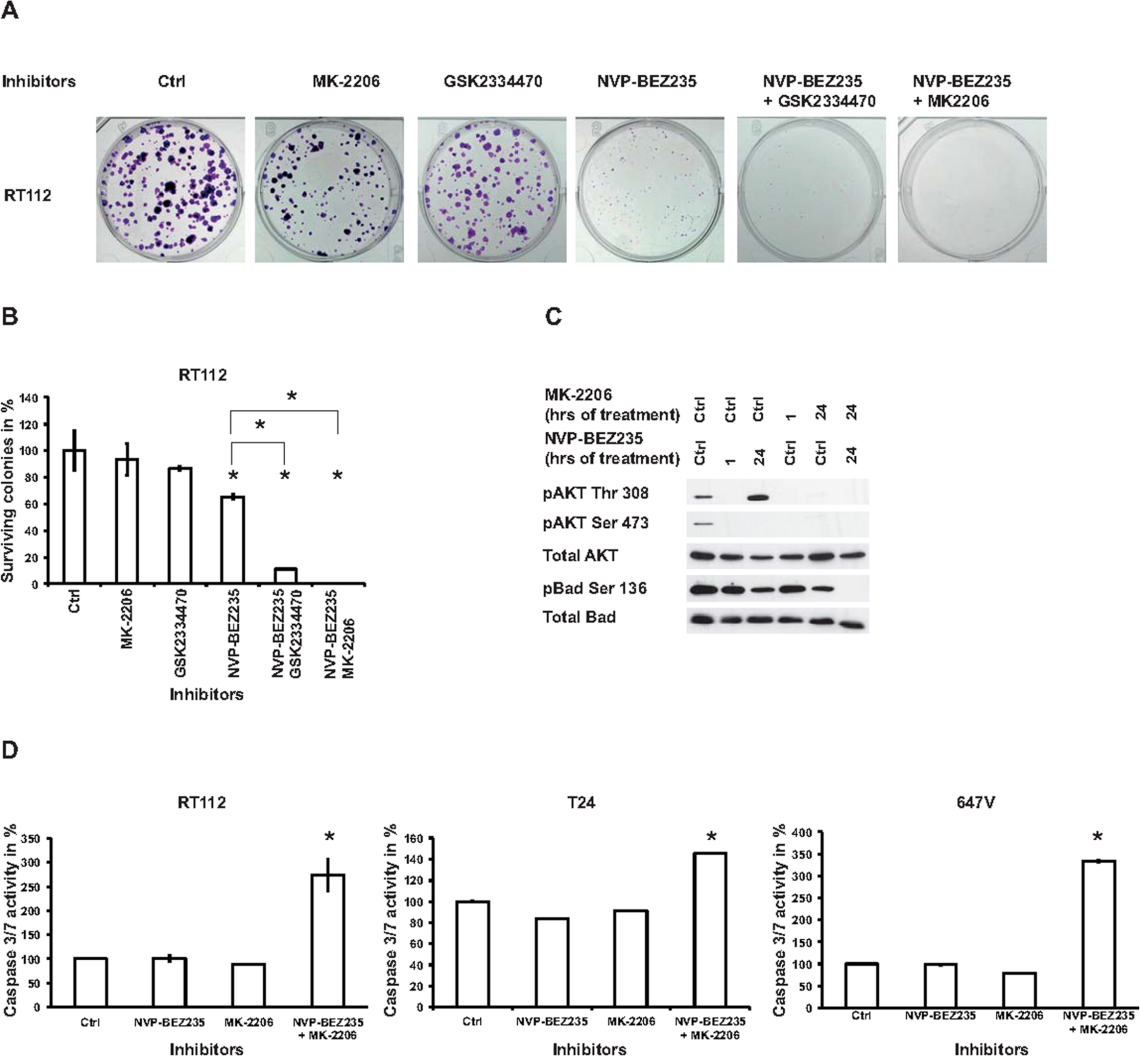
The combination of dual PI3K and AKT inhibitors regulate colony formation and apoptosis. (A) RT112 cells were treated with control (ctrl), 1000 nM MK-2206, 500 nM GSK2334470, 200 nM NVP-BEZ235 or their combinations for 12 days and colonies were stained with 0.5% crystal violet. (B) Quantification of surviving colonies in duplicate samples relative to control expressed as a percentage and three independent experiments. (C) Cells were lysed and immunoblotting was performed with the indicated antibodies after treating cells with the respective inhibitors or control (ctrl) for the indicated time points. Results are representative of at least three independent experiments. (D) RT112, T24 or 647V cells were treated with 200 nM, 100 nM and 200 nM NVP-BEZ235 respectively, 1000 nM MK-2206, their combination or control (ctrl) for 48 hours and caspase 3/7 assay was performed. Results obtained in arbitrary luminescence units were normalized to the number of living cells per trypan blue staining conducted in parallel and indicate the mean +/- standard error of three independent experiments relative to control expressed as a percentage. * indicates p < 0.05 (unpaired Student’s T-test.

In order to translate these results to long-term survival we used colony formation assays. Colony size decreased dramatically after NVP-BEZ235 treatment but 65% colonies continued to survive Fig 4A and 4B.

No significant effects were observed using MK-2206 or GSK2334470, whereas combining NVP-BEZ235 with either GSK2334470 or MK-2206 almost completely suppressed colony formation.

The combination of MK-2206 and NVP-BEZ235 suppressed AKT hyperphosphorylation 24 hours after treatment and induced apoptosis measured by dephosphorylated Bad and caspase 3/7 activity Fig 4C and 4D. Compared to NVP-BEZ235 monotherapy, cell cycle progression was not further affected S6A Fig. The combination index (CI) theorem demonstrated in cell viability assays that both combinations were synergistic with CI values ranging from 0.08 to 0.87 S6B Fig and S1 Table. Dose reduction indices (DRI) for NVP-BEZ235 were greater than 1, indicating that a comparable anti-tumor effect requires lower concentrations of NVP-BEZ235 when combined with either PDK1 or AKT inhibitors.

### Combination treatment of BEZ-235 and MK2206 strongly reduces tumor growth

**Fig 5:**
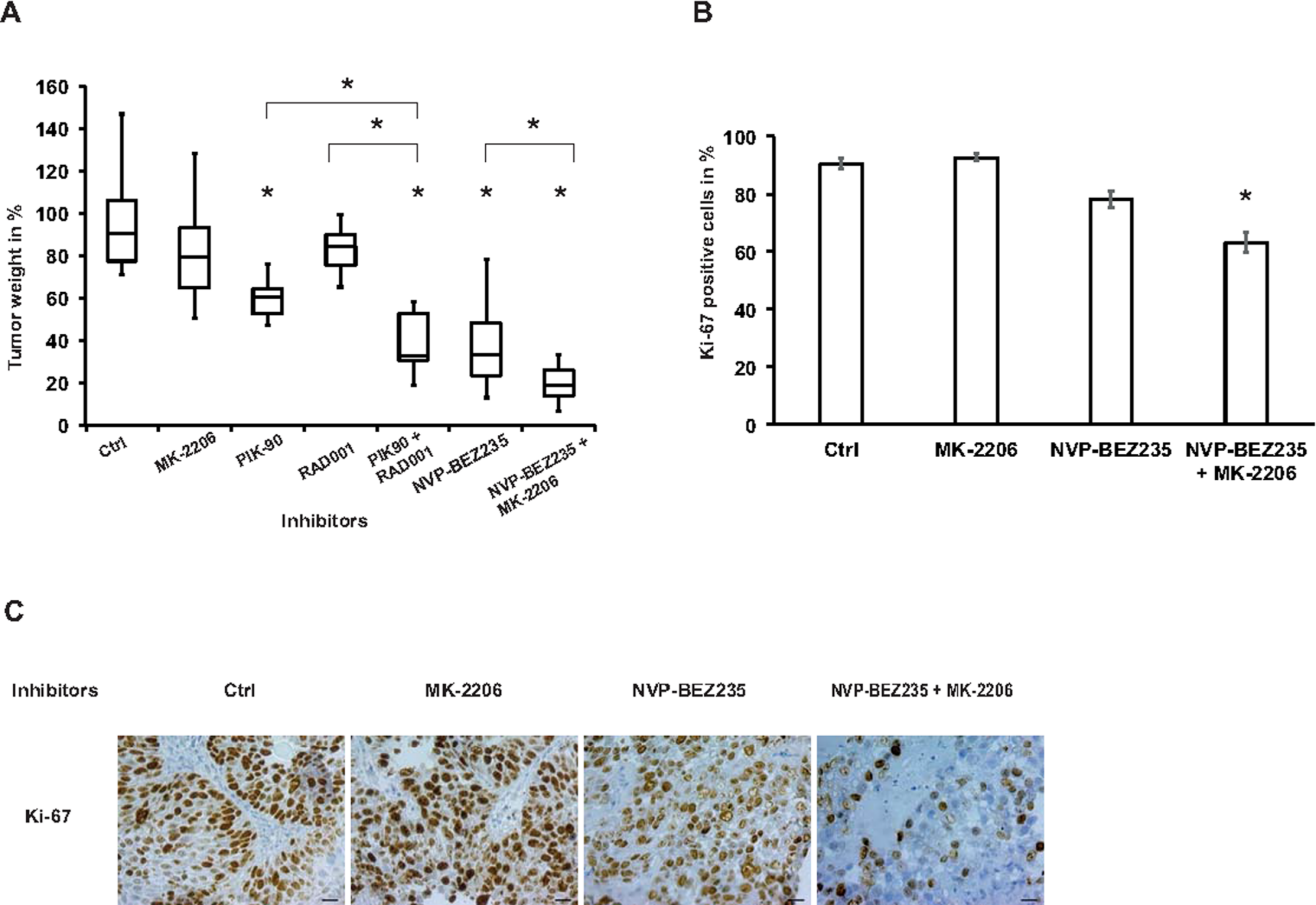
Multiple targeting of the PI3K signaling pathway inhibits 3-dimensional tumor growth. RT112 cells were grown on the CAM as xenografts and were treated with 1000 nM MK-2206, 2000 nM PIK-90, 5 nM RAD001, 200 nM NVP-BEZ235, their indicated combinations or control (ctrl). (A) Tumor weight shown as percentage of control from 7–21 tumors per condition in two independent experiments. Horizontal line indicates median, upper whisker indicates the difference between maximum and first quartile, and lower whisker indicates the difference between minimum and third quartile. * indicates p < 0.05. (B) Ki-67 positive cells were quantified from three fields from at least three tumors treated with the indicated inhibitors and expressed as a percentage. * indicates p < 0.05 (unpaired Student’s T-test). (C) Representative images of tumor sections treated with respective inhibitors and stained with Ki-67 antibody by immunohistochemistry. Scale bar indicates 20 um.

For translation the in vitro observations into a three-dimensional tumor xenograft system we applied the chicken chorioallantoic membrane (CAM) mode [34]. Treatment of tumors with either PIK-90 or RAD001 resulted in decreases in tumor weight of 35% or 20% respectively with no effect of MK-2206 Fig 5A.

Combining PIK-90 with RAD001 or using NVP-BEZ235 resulted in a 60% decrease. However, combination of NVP-BEZ235 with MK-2206 resulted in an additional 46% reduction in tumor weight as compared to treatment with NVP-BEZ235 alone. The level of Ki-67 expression was not reduced by MK-2206 but decreased by 13% with NVP-BEZ23 and 30% when combining NVP-BEZ235 and MK-2206 Fig 5B and 5C [24].

## Discussion

Understanding the complexity of the PI3K signaling pathway is a crucial prerequisite for designing therapies that overcome the present limitations in clinical application. One of the challenges is to understand the cellular response to a specific drug in a tumor entity in order to understand what kind of feedback loops as cellular rescue mechanisms are induced after treatment.

We have previously demonstrated that various classes of inhibitors against molecules in the PI3K pathway differ in their effects on S6K1 and 4E-BP1 phosphorylation level. Understanding the regulation of 4E-BP1 phosphorylation is imperative since its inhibition is required to mediate an effective reduction in cell growth and proliferation [35]. Our previous results and the present study demonstrate that the inhibition of either PI3K, AKT, mTORC1 or mTOR alone is insufficient to reduce 4E-BP1 phosphorylation in most cell lines examined, whereas dual PI3K/mTOR inhibitors successfully suppress 4E-BP1 phosphorylation [23,24]. Regulation of 4E-BP1 phosphorylation by parallel inactivation of both PI3K and mTOR is supported by the concentration dependency of this effect with PI3K inhibitors, PIK-90 and BKM-120, the dual PI3K/mTOR inhibitor NVP-BEZ235 as well as a combination of PIK90 and the mTORC1 inhibitor RAD001. We extended these data by combining the PI3K inhibitor PIK-90 with either mTOR shRNA, Raptor or Rictor siRNA. These data indicate that concomitant inhibition of PI3K and mTORC1, but not mTORC2, results in 4E-BP1 dephosphorylation. Furthermore, AKT inhibition failed to affect 4E-BP1 phosphorylation, alone or in combination with Raptor silencing.

**Fig 6:**
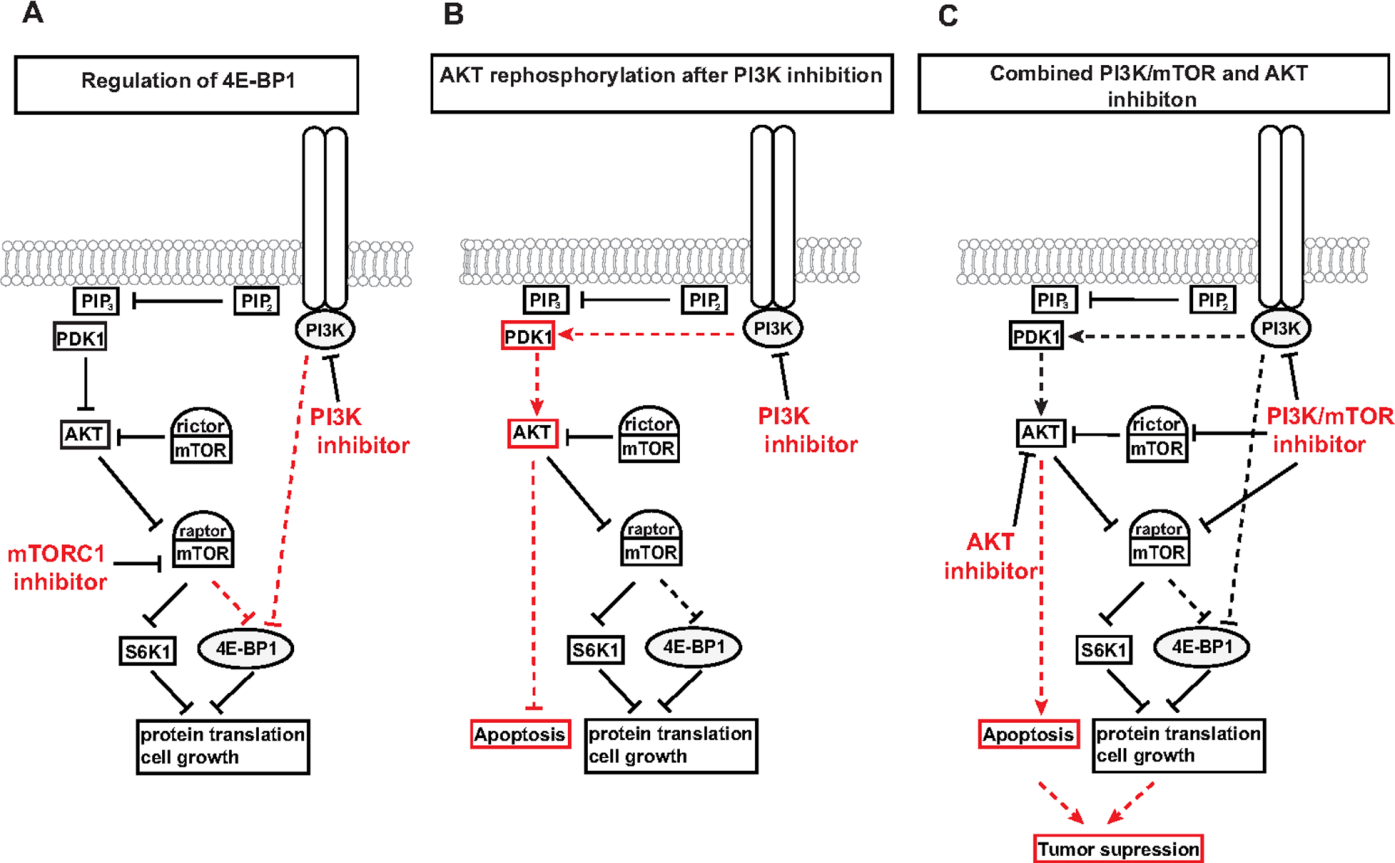
Schematic representation of the effects of PI3K pathway inhibitors in BLCA. (A) For successful therapy response, dual mTOR and PI3K inhibition is required to inhibit 4E-BP1 activity (B) Inhibition of PI3K leads to PDK1-mediated AKT rephosphorylation that does not require PIP3 and mTORC2 (C) Combination therapy with dual PI3K/mTOR and AKT inhibitors results in AKT and 4E-BP1 dephosphorylation, induction of apoptosis and reduction of cell growth to enable effective tumor supression.

It has been proposed that the lack of 4E-BP1 dephosphorylation observed with allosteric mTORC1 inhibitors like rapalogs can be overcome by using ATP-competitive mTOR inhibitors [36]. Using INK128 as an example of this class of mTOR inhibitors, we show here that only at non-specific doses that besides mTORC1 additionally inhibit PI3K-AKT induce inhibitory effects on 4E-BP1. Raptor regulates the catalytic activity of mTOR by controlling the recognition and recruitment of substrates via their TOS motifs [8,37,38]. Moreover, Raptor-free mTOR is capable of phosphorylating S6K1 but not 4E-BP1, providing a basis for the differential regulation of these two substrates by mTORC1[39]. Our results support this notion and indicate that the regulation of 4E-BP1 by mTOR relies on the gatekeeper function of Raptor. However, silencing either Raptor or mTOR expression alone did not reduce 4E-BP1 phosphorylation and required additional PI3K inhibition. Hence, PI3K may influence Raptor function, directly or indirectly. Of note, the combined inhibition of Raptor and PI3K dephosphorylated 4E-BP1 more completely than inhibition of mTOR and PI3K. Taken together, we postulate that formation of the mTORC1 protein complex rather than regulation of mTOR kinase activity might be controlled by PI3K activity by a mechanism that is independent from the PI3K/AKT axis and which is a crucial step in the control of 4E-BP1 activation. Interestingly, dephosphorylation of S6K1 was observed using inhibitors of PI3K, AKT, PDK1 and also mTOR or mTORC1. This indicates that S6K1 is regulated by mTORC1 and that in the context of bladder cancer, inhibitors directed against upstream elements of the pathway do not activate feedback elements such as GSK1 that can rescue mTORC1 activity as described in other tumor entities[14]. Our results demonstrate a novel mechanism for the regulation of 4E-BP1 phosphorylation in bladder cancer, which is mediated by PI3K and mTORC1 but is not dependent on activity of AKT Fig 6A.

From a translational perspective, we have also demonstrated that dual PI3K/mTOR inhibitors result in a greater reduction in cell viability as compared to either PI3K, AKT or mTORC1 inhibitors alone Fig 1E and S4B Fig [24]. Previous reports investigating PI3K pathway inhibition in bladder cancer have demonstrated that cells possessing activating mutations in PIK3CA are selectively sensitive to PI3K or AKT inhibition, while TSC1 or mTOR mutations contribute to sensitivity to rapalogs [40–42]. Thus, monotherapy with those inhibitors is therefore only suitable for a highly selected patient population and final parameter for stratification have not been validated to date. We therefore investigated combination therapies to improve the efficacy in patients. The panel of cell lines included in our study possesses diverse molecular alterations in FGFR3, RAS, PIK3CA, PTEN, TSC1 and mTOR that are capable of influencing response to PI3K pathway inhibition and are very frequent in bladder cancer specimen [24,43,44]. Our results indicate that bladder cancer cells are sensitive to dual PI3K/mTOR inhibitors regardless of their genetic background. Furthermore, this response is greater than that achieved with PI3K, AKT, mTOR or PDK1 inhibitors alone.

Despite the promising effects of dual PI3K/mTOR inhibitors in cell lines, a recent clinical trial failed to demonstrate clinical benefit with NVP-BEZ235 as second line therapy in bladder cancer patients with locally advanced or metastatic disease [45]. In this trial, stable disease or partial response were observed in 3 out of 20 patients. NVP-BEZ235 also demonstrated an unfavorable toxicity profile with 10 patients experiencing grade 3–4 adverse effects. A similar pattern of clinical efficacy and toxicity has been demonstrated in clinical trials with other tumor entities [12,46].

This failure may be explained in part by our observation that the efficacy of dual PI3K/mTOR inhibitors is limited by a feedback activation of AKT over time. It has previously been described that the p110β isoform results in a PIP3 dependent compensatory AKT phosphorylation following selective p110α inhibition [15]. We demonstrate here a novel feedback activation of AKT that occurs despite suppression of all PI3K isoforms, requires additional suppression of PDK1 activity and occurs independently of PIP3 synthesis, revealing an additional level of adaptive signaling after PI3K inhibition. Similar to previously published data, PDK1 inhibition alone by GSK2334470 was insufficient to control AKT dephosphorylation [14]. Our results indicate that this requires additional inhibition of PI3K Fig 3 and S4 and S5 Figs. We could also observe previously described AKT rephosphorylation following treatment with mTOR kinase inhibitors that is proposed to depend on feedback activation of receptor tyrosine kinases and formation of PIP3 [47]. In agreement with published results, this rephosphorylation could be partially suppressed by additional PDK1 inhibition for one hour [33]. However, at 24 hours this combination was insufficient to suppress AKT phosphorylation.

Our data indicate that this is due to persistence of PI3K activity and demonstrate a tight interplay between mTOR, PI3K and PDK1 that can induce AKT rephosphorylation surprisingly also independently of PIP3. The key molecule for maintaining phosphorylated AKT despite concurrent mTOR and PDK1 inhibition seems to be PI3K. This suggests a novel link between PI3K and AKT, independent of PIP3 dependent PDK1 regulation of AKT phosphorylation Fig 6B. Previously described PIP3 independent mechanisms of PDK1 mediated regulation of AKT might play a role in this process and raise the intriguing possibility that PI3K controls these processes by regulating protein complex formation [33,48]. Hence, if AKT should not be targeted directly, for sustained dephosphorylation of AKT both PI3K and PDK1 must be simultaneously inhibited. These compensatory signaling mechanisms might operate to ensure the persistence of activated AKT to enable or maintain cell proliferation in the presence of inhibitory signals.

AKT hyperphosphorylation resulting from dual PI3K/mTOR inhibitors was suppressed by a combination treatment with AKT or PDK1 inhibitors. This combination was synergistic and resulted in apoptosis induction, reduced colony formation ability and decreased tumor growth, as compared to dual PI3K/mTOR inhibition alone Fig 6C. The combination therapy also demonstrated DRI values greater than 1, which might permit a reduction in the dose of NVP-BEZ235 to avoid its dose-dependent toxicities. The combined inhibition of PI3K, AKT and mTOR ensures the dephosphorylation of the essential downstream targets S6K1, 4E-BP1 and AKT and is required for an effective and sustained reduction in tumor growth. This pre-clinical study provides an explanation for the limitations of the efficacy of targeting the PI3K pathway in the clinic. We also present a novel treatment regimen that might benefit a broad range of patients and could limit dose-dependent toxicities.

## Conclusion

The PI3K signaling pathway is frequently altered in cancer and thus provides an attractive target for cancer therapy. However, inhibitors against this pathway showed very disappointing results in clinical trials. We present a comprehensive analysis of functional and biochemical effects using different classes of inhibitors to understand limitations and improve therapy designs used to date. Our results demonstrate that PI3K/mTORC1 regulate 4E-BP1 phosphorylation independent from AKT. Long-term inhibition of PI3K results in PDK1 mediated rephosphorylation of AKT. Simultaneous targeting of PI3K, AKT and mTOR is required for effective tumor suppression by promoting sustained AKT, S6K1 and 4E-BP1 dephosphorylation and induction of apoptosis. Since this therapy acts synergistic it might reduce adverse effects as well as improve patient response.

## Supporting information

S1 Fig Effect of RAD001 on multiple bladder cancer cell lines. Cells were treated with RAD001 at the indicated concentrations for 1 hour and immunoblotting was performed on lysates with the denoted antibodies. Results are representative of at least three independent experiments.
(TIF)

S2 Fig Effect of PIK90 and BKM-120 on multiple bladder cancer cell lines. Cells were treated with respective inhibitors at the indicated concentrations for 1 hour and immunoblotting was performed on lysates with the denoted antibodies for PIK90 (A) and BKM-120 (B). Results are representative of at least three independent experiments.
(TIF)

S3 Fig Effect of PI3K, mTOR, and other Kinase inhibitors on multiple bladder cancer cell lines. Cells were treated with respective inhibitors at the indicated concentrations for 1 hour and immunoblotting was performed on lysates with the denoted antibodies for NVP-BEZ235 (A), BKM-120 (B) and KU60019, NU7441 and GSK2334470 (C). Results are representative of at least three independent experiments.
(TIF)

S4 Fig Combination of PI3K and mTORC1 inhibition on multiple bladder cancer cell lines. Cells were treated with respective inhibitors at the indicated concentrations (A) for 1 hour and immunoblotting was performed on lysates with the denoted antibodies, (B) for 72 hours and cell viability assay was performed. Results indicate the mean +/- standard error of relative cell fluorescence in arbitrary units expressed as a percentage of control from three independent experiments.
(TIF)

S5 Fig PI3K and PDK1 inhibition results in positive feedback loops of AKT phosphorylation. (A) T24 and 647V cells were treated with 100 nM or 200 nM NVP-BEZ235 respectively for the indicated duration or with control (ctrl). Cells that were treated for 24 and 48 hours were additionally retreated with the same concentration for 1 hour (indicated by +, no retreatment indicated by -) and immunoblotting was performed with the respective antibodies (B) NVP-BEZ235 or control treatment was combined with 500 nM of GSK2334470 for the indicated duration or with control (ctrl). Immunoblotting was performed with the indicated antibodies. Results are representative of at least three independent experiments. (C to E) Cells were treated with control (ctrl), 500 nM GSK2334470, 500 nM PIK-90, 25 nM INK128 or 10 nM NVP-BEZ235 for the indicated duration and immunoblotting was performed on lysates with the indicated antibodies. Results are representative of at least three independent experiments.
(TIF)

S6 Fig Effects of BEZ-235 and MK-2206 on cell cycle progression and cell viability. (A) Cells treated with 200 nM NVP-BEZ235, 1000 nM MK-2206, their combination or control for 24 hours were labeled with EdU and 7-AAD and the cell cycle distribution was analyzed. Results indicate the mean +/- standard deviation of percentage of total cells in the respective cell cycle phases and are representative of two independent experiments. * indicates p < 0.05. (B) RT112, T24 or 647V cells were treated with indicated increasing concentrations of BEZ235, 1000 nM MK-2206, 500 nM GSK2334470, their indicated combinations or with control (ctrl) for 72 hours and cell viability assay was performed. Results indicate the mean +/- standard error of relative cell fluorescence in arbitrary units expressed as a percentage of control from three independent experiments.
(TIF)

S1 Table Effects of combination therapy on cell viability using increasing concentrations of NVP-BEZ235 with 500 nM GSK2334470 or 1000 nM MK-2206 were assessed using the combination index theorem. For each combination, the combination index (CI) and dose reduction index (DRI) were determined. All analysis was conducted on the average values from three independent experiments, depicted in S6B Fig.
(EPS)
