# Supplementary figures and images for: Parallel PI3K, AKT and mTOR inhibition is required to control feedback loops that limit tumor therapy
Source: PLoS One. 2018 Jan 22;13(1):e0190854. doi: 10.1371/journal.pone.0190854 (PMC5777650; doi:10.1371/journal.pone.0190854)

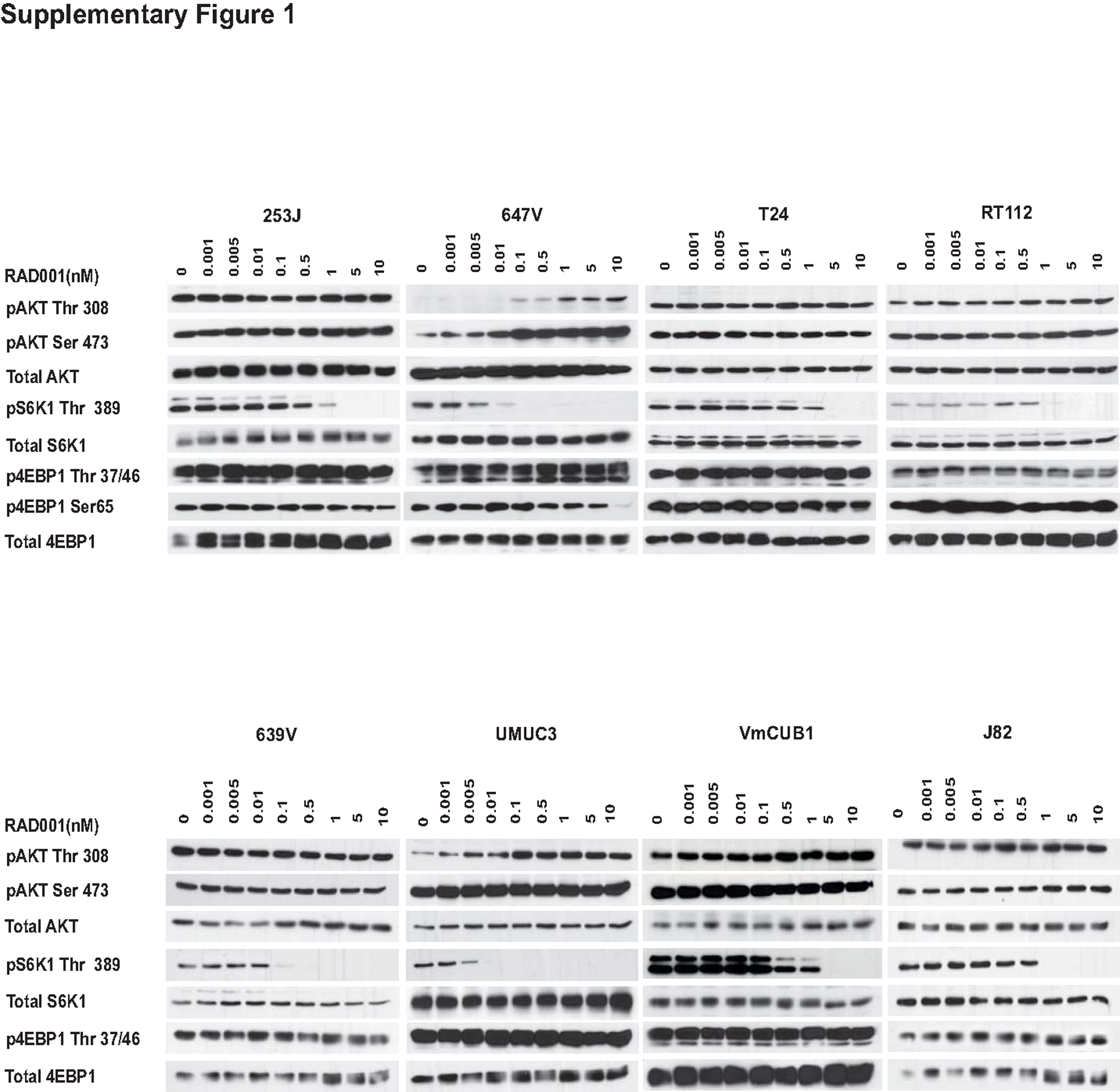

Supplement: S1 Fig — Cells were treated with RAD001 at the indicated concentrations for 1 hour and immunoblotting was performed on lysates with the denoted antibodies. Results are representative of at least three independent experiments. (TIF) [file pone.0190854.s001.tif]

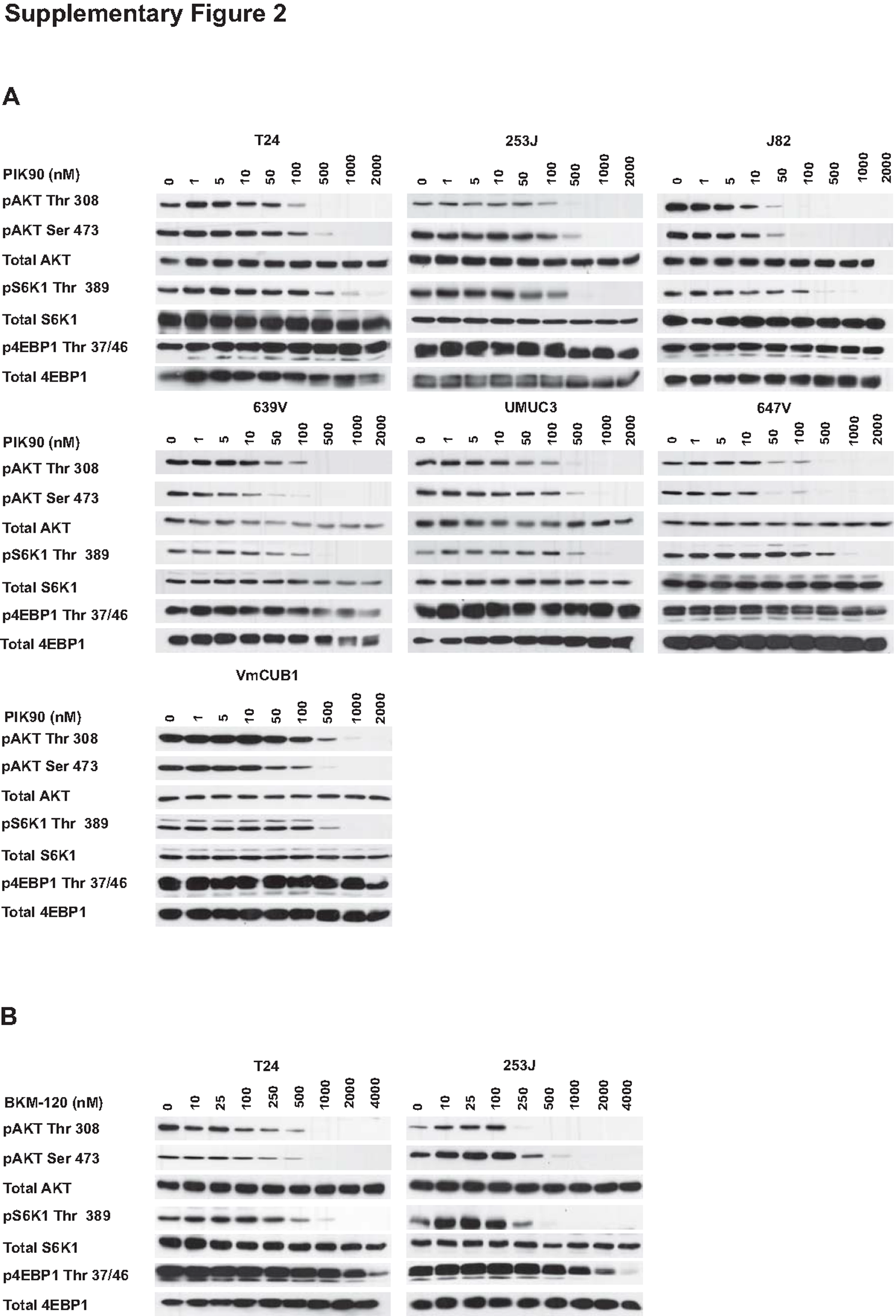

Supplement: S2 Fig — Cells were treated with respective inhibitors at the indicated concentrations for 1 hour and immunoblotting was performed on lysates with the denoted antibodies for PIK90 (A) and BKM-120 (B). Results are representative of at least three independent experiments. (TIF) [file pone.0190854.s002.tif]

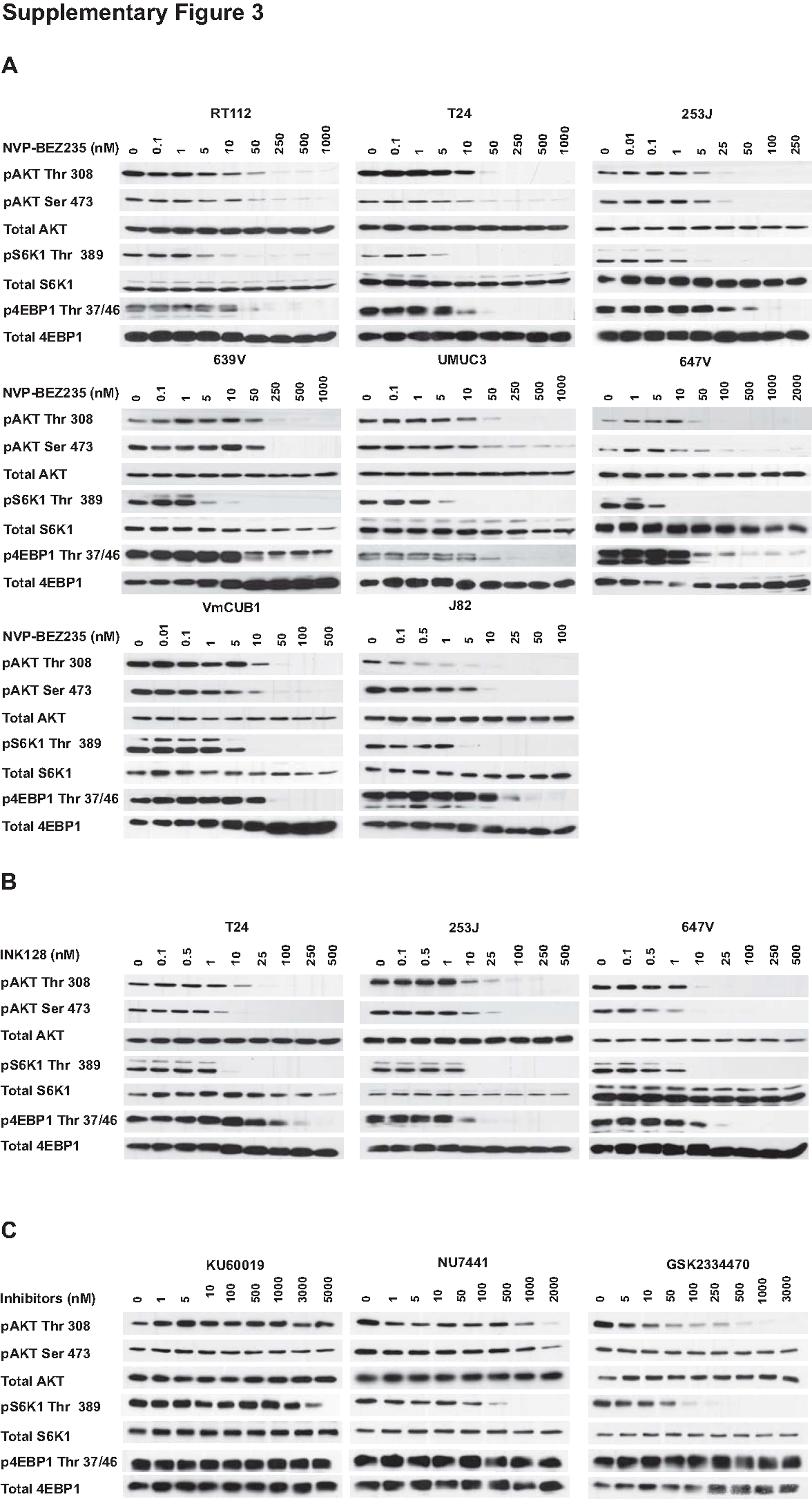

Supplement: S3 Fig — Cells were treated with respective inhibitors at the indicated concentrations for 1 hour and immunoblotting was performed on lysates with the denoted antibodies for NVP-BEZ235 (A), BKM-120 (B) and KU60019, NU7441 and GSK2334470 (C). Results are representative of at least three independent experiments. (TIF) [file pone.0190854.s003.tif]

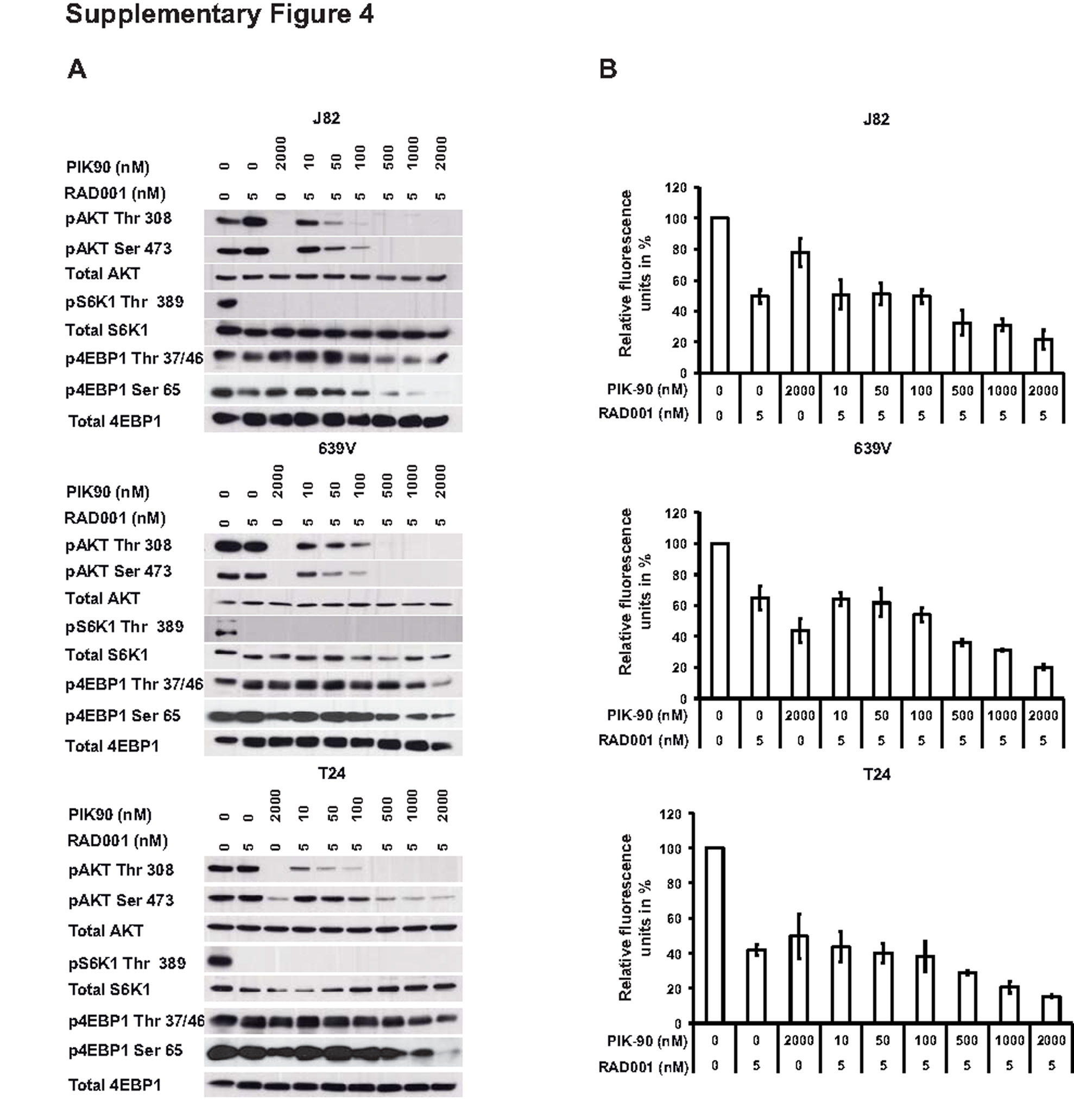

Supplement: S4 Fig — Cells were treated with respective inhibitors at the indicated concentrations (A) for 1 hour and immunoblotting was performed on lysates with the denoted antibodies, (B) for 72 hours and cell viability assay was performed. Results indicate the mean +/- standard error of relative cell fluorescence in arbitrary units expressed as a percentage of control from three independent experiments. (TIF) [file pone.0190854.s004.tif]

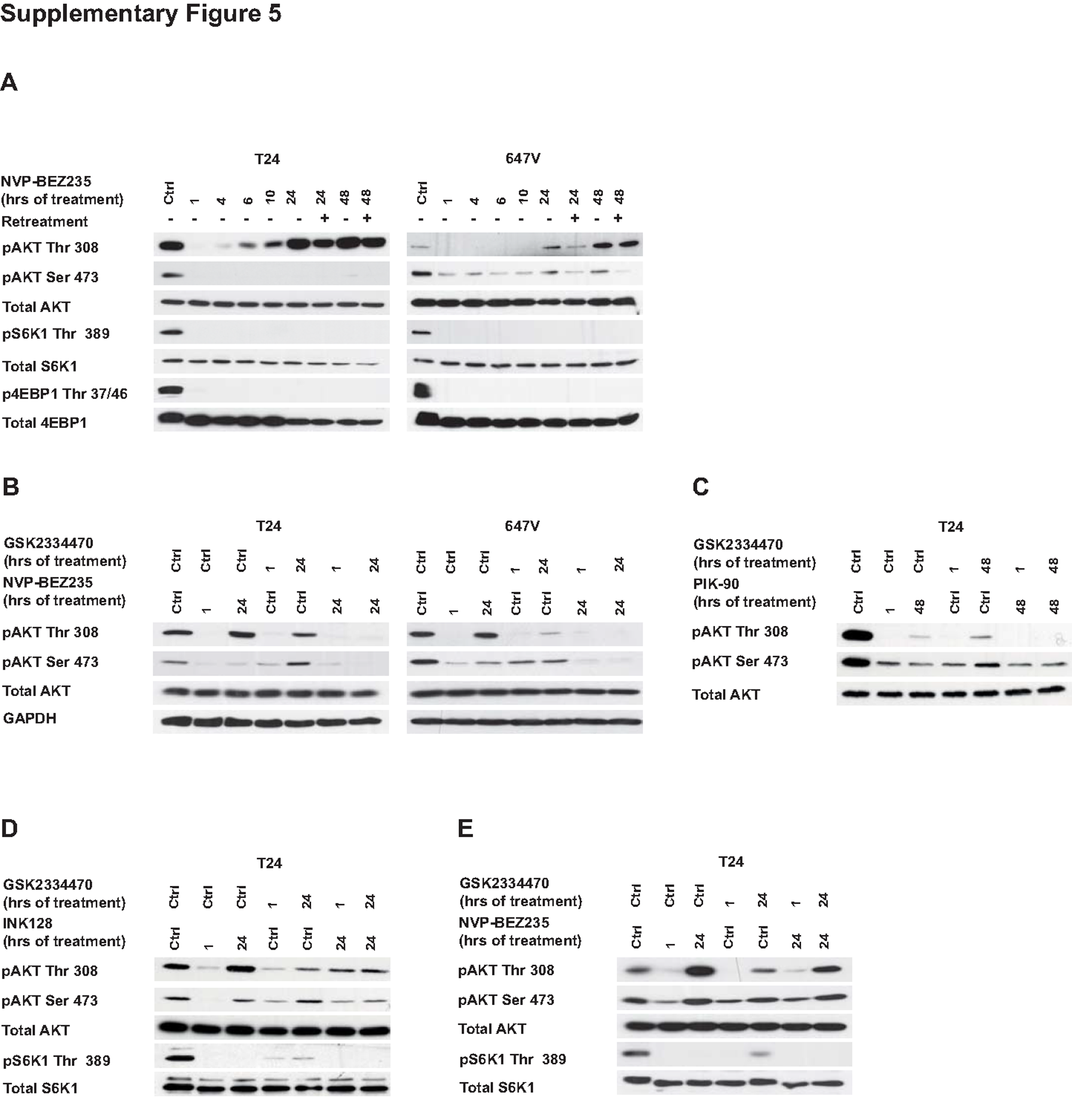

Supplement: S5 Fig — (A) T24 and 647V cells were treated with 100 nM or 200 nM NVP-BEZ235 respectively for the indicated duration or with control (ctrl). Cells that were treated for 24 and 48 hours were additionally retreated with the same concentration for 1 hour (indicated by +, no retreatment indicated by -) and immunoblotting was performed with the respective antibodies (B) NVP-BEZ235 or control treatment was combined with 500 nM of GSK2334470 for the indicated duration or with control (ctrl). Immunoblotting was performed with the indicated antibodies. Results are representative of at least three independent experiments. (C to E) Cells were treated with control (ctrl), 500 nM GSK2334470, 500 nM PIK-90, 25 nM INK128 or 10 nM NVP-BEZ235 for the indicated duration and immunoblotting was performed on lysates with the indicated antibodies. Results are representative of at least three independent experiments. (TIF) [file pone.0190854.s005.tif]

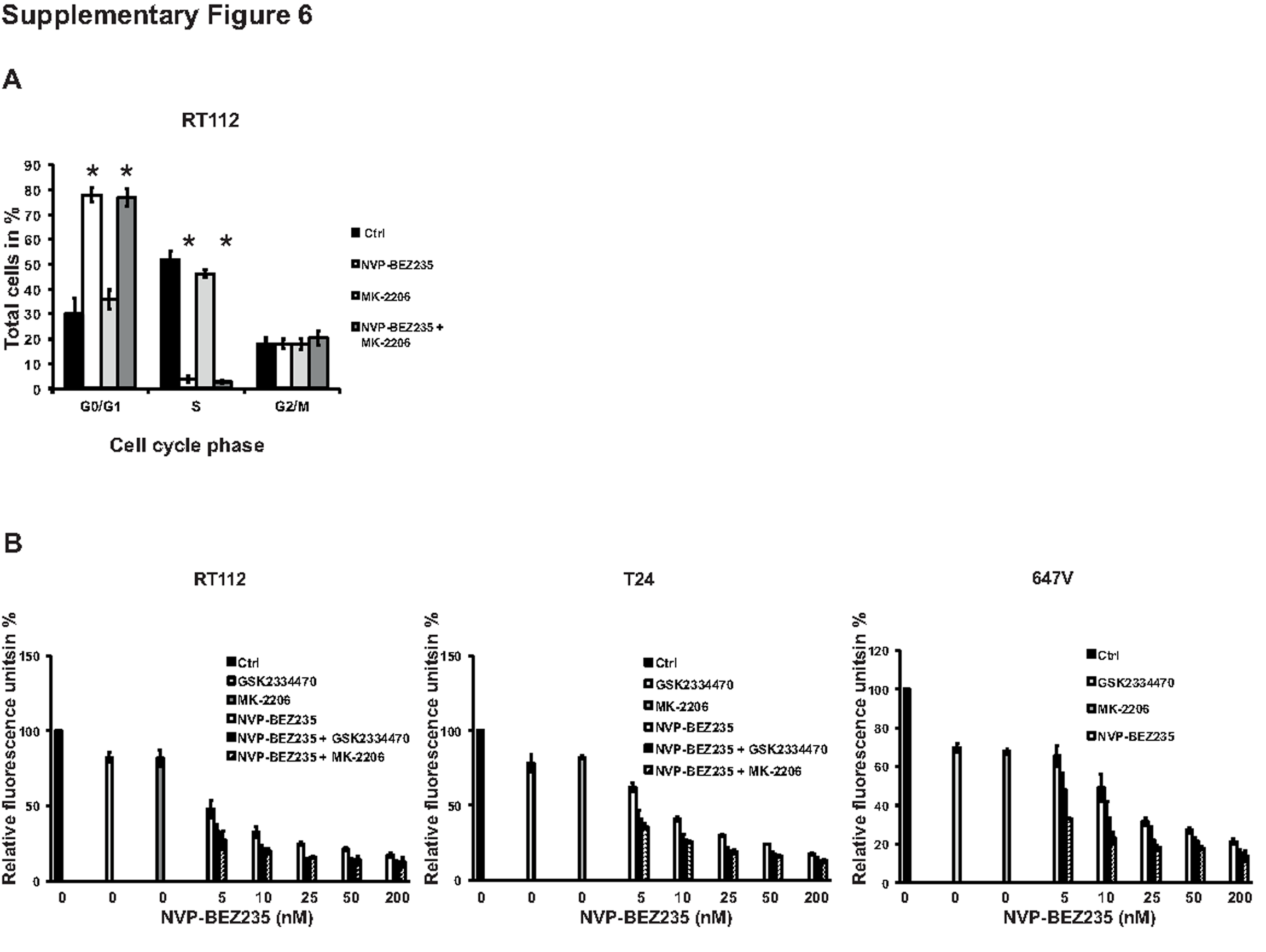

Supplement: S6 Fig — (A) Cells treated with 200 nM NVP-BEZ235, 1000 nM MK-2206, their combination or control for 24 hours were labeled with EdU and 7-AAD and the cell cycle distribution was analyzed. Results indicate the mean +/- standard deviation of percentage of total cells in the respective cell cycle phases and are representative of two independent experiments. * indicates p < 0.05. (B) RT112, T24 or 647V cells were treated with indicated increasing concentrations of BEZ235, 1000 nM MK-2206, 500 nM GSK2334470, their indicated combinations or with control (ctrl) for 72 hours and cell viability assay was performed. Results indicate the mean +/- standard error of relative cell fluorescence in arbitrary units expressed as a percentage of control from three independent experiments. (TIF) [file pone.0190854.s006.tif]
